# Efficacy of orally administered fluralaner in dogs against laboratory challenge with *Haemaphysalis longicornis* ticks

**DOI:** 10.1186/s13071-019-3306-1

**Published:** 2019-01-18

**Authors:** Masanori Toyota, Kyoko Hirama, Tatsumi Suzuki, Rob Armstrong, Tatsuyuki Okinaga

**Affiliations:** 1 R&D, Intervet K.K, 1-13-12 Kudan-kita, Chiyodak-ku, Tokyo, Japan; 2 Central Research Laboratory, Intervet K.K, 1103 Fukaya, Kasumigaura, Ibaraki Japan; 3 Merck Animal Health, 2 Giralda Farms, Madison, NJ USA

**Keywords:** *Haemaphysalis longicornis*, Dogs, Fluralaner

## Abstract

**Background:**

*Haemaphysalis longicornis* ticks represent an ectoparasitic health threat to dogs. This study evaluated the immediate and persistent efficacy of orally administered fluralaner for control of this tick.

**Methods:**

Twenty-four dogs were sorted into 4 groups based on their tick carrying capacity measured in a preliminary challenge. Two days before treatment, dogs were challenged with *Haemaphysalis longicornis* and then at the time of treatment dogs received with oral fluralaner at 10, 25 or 50 mg/kg respectively to 3 of the groups, while the remaining group was sham treated. Ticks were counted and categorized on all dogs 2 days after treatment (4 days after challenge). Tick challenges were repeated at 28, 56, 84 and 112 days following treatment with tick counts 48 hours following each challenge. Tick control efficacy was evaluated by comparing the mean (geometric) total live attached and dead engorged ticks on each fluralaner treated group with the sham treated dogs.

**Results:**

Oral fluralaner is highly acaricidal for *H*. *longicornis* that feed on treated dogs. The mean efficacy rate in dogs treated with fluralaner at the commercial dose range of 25 to 50 mg/kg was greater than 90% at 114 days after treatment, whereas efficacy at this time in dogs treated at 10 mg/kg was 79%.

**Conclusions:**

Fluralaner administered orally to dogs within the commercial dose range at 25 to 50 mg/kg is effective for up to 114 days against laboratory challenge with *H*. *longicornis* ticks.

## Background

*Haemaphysalis longicornis* is an ixodid tick common in temperate areas particularly in Asian countries [1, 2]. There is evidence that the geographic range of this tick has increased, spreading to new countries around Southeast Asia and more recently the tick has been confirmed in the USA [3]. *Haemaphysalis longicornis* can reproduce through parthenogenesis [4] and tick larvae have been found infesting birds, contributing to this tick’s ability to expand its geographical range. *Haemaphysalis longicornis* infests multiple species, including dogs [5] and humans [6]. It is also known to transmit multiple pathogens including *Anaplasma* spp., *Rickettsia* spp., *Babesia* spp., as well as severe fever with thrombocytopenia syndrome virus (SFTSV) [7].

Fluralaner (Bravecto Chew, MSD Animal Health, Madison, NJ, USA) is a systemically distributed isoxazoline class insecticide and acaricide that delivers persistent high efficacy against ticks, including adult *Ixodes ricinus*, for 12 weeks following oral administration in dogs of the commercial dose of 25 to 56 mg/kg [8]. This persistent efficacy improves dog owner compliance with veterinarian recommendations for ectoparasite control [9]. The hypothesis tested in this study is that oral fluralaner administration to dogs will deliver persistent efficacy against *H*. *longicornis* infestation.

## Methods

Parthenogenetic *H*. *longicornis* ticks (Okayama strain) were cultured on rabbits for use in this blinded and negative controlled study. Facility temperature was held between 20–25 °C, while artificial and natural light were used to consistently provide 12 hours daylight. All participating 6–7 month-old Beagle dogs were clinically examined by a veterinarian and confirmed to be healthy; bathed with non-medicated commercially available shampoo and then acclimatized to the study site for 7 days. Participating dogs were housed individually in isolator cages with a toy and daily social interaction for environmental enrichment. Dogs were fed a commercial dry diet once daily with free access to water at all times.

The dogs were ranked within their sex group according to descending tick carrying rate in an initial challenge conducted 7 days before treatment administration with tick counts 48 hours later. For all tick challenges and subsequent counting, dogs were sedated with 5 mg/kg ketamine and 2 mg/kg xylazine administered by intramuscular injection. Dogs were then randomly sorted into 4 groups of 6 dogs based on their tick carrying ranking and using computer-generated randomization. Any dogs with identical carrying rates were sub-ranked by ascending ID number. Each group received one of 4 different treatments: untreated, 10, 25, or 50 mg fluralaner/kg body weight. The specific fluralaner dose was prepared based on each dog’s body weight and by administering the appropriate quantity of 13.64% fluralaner medicated chewable tablets. All dogs were observed at 1 h intervals for 4 h following treatment to confirm that the medication was not regurgitated and to document any potential treatment related adverse events. Study personnel conducting veterinary examinations, general observations, tick infestations and tick counting were blinded to the treatment status.

Tick infestation challenges [10] were conducted on all groups 2 days before the treatment day, and 28, 56, 84 and 112 days following treatment administration. For the challenge, 25 viable unfed adult *H*. *longicornis* were placed in 5 different locations on each dog: ears (5 ticks on each ear) and caudal trunk (5 ticks on each of the right side, center and left side). The number of sites was selected to give each tick enough space to achieve high infestation rates, and ticks were held at the site in a perforated plastic Petri dish (60 × 15 mm) that was attached to the selected site on the dog with medical adhesive. Breathable mesh was placed between the dish and its perforated lid and dishes were covered and firmly held in place on the dogs using breathable bandage and tape. Dogs were observed regularly throughout the 48 hour challenge to confirm that the apparatus remained in place at the selected location. If there was any indication that the bandage and/or tape was loosening then the investigator re-fixed the apparatus in place using new bandage and tape.

Ticks were counted and categorized using criteria previously described [11] either 4 days after challenge (2 days after treatment), or 48 h after challenge at 30, 58, 86 and 114 days following treatment administration. To conduct the counts, the bandage and tape were removed from the sedated dogs and the tick retaining apparatus lid was opened. The attachment status category and viability of all ticks in the apparatus was recorded [11].

A tick control efficacy rate (%) was calculated for each study group of dogs at each time point using the formula:

$$ \mathrm{Efficacy}\left(\%\right)=\left(\mathrm{MC}\hbox{-} \mathrm{MT}\right)/\mathrm{MC}\times 100 $$

where MC is the mean (geometric) live attached ticks on control group dogs and MT is the mean (geometric) live attached ticks on dogs in fluralaner-treated dogs.

Treatment at each time point was declared to be effective when the calculated efficacy exceeded 90%.

## Results and discussion

No adverse events were reported after drug administration, and no abnormal clinical signs were observed throughout the entire study duration. The mean infestation rate in the negative control animals was 98.7%, 99.3%, 98.0%, 99.3% and 98.0% at 2, 30, 58, 86 and 114 days after treatment respectively, which is > 95% at every time point and indicates an adequate tick challenge. Calculated geometric mean efficacy rates for each time period (Table 1) show that treatment was effective (> 90% efficacy) at every time point except for 114 days following treatment at a dose of 10 mg/kg. There were no differences in efficacy between the five tick attachment sites (data not shown).

**Table 1:** Calculated *Haemaphysalis longicornis* mean (geometric) efficacy (%) in dogs administered one of three different orally administered fluralaner doses and compared with untreated control dogs

Days post-treatment	Oral fluralaner dose		
	10 mg/kg	25 mg/kg	50 mg/kg
2	100	100	100
30	100	100	100
58	97.9	99.5	100
86	95.4	99.5	96.9
114	79.0	93.6	95.9

A single oral fluralaner treatment administered to dogs at 25–50 mg/kg is an effective systemic acaricide against *H*. *longicornis* for up to 114 days following treatment. This treatment modality provides a simple approach for providing dog owners with an extended duration of protection against this tick. Dogs in the untreated group consistently maintained a tick infestation rate greater than 95% for at least 2 days following each challenge, confirming that this was an adequate challenge. The efficacy rate observed in dogs treated orally with fluralaner at 10 mg/kg was 79% at 114 days after treatment which is below the pre-determined threshold for efficacy. This dose is also below the standard commercial dose of fluralaner which is 25–56 mg/kg. These results extend the efficacy duration observed following a single dose of another isoxazoline, lotilaner, which was effective against *H*. *longicornis* for 37 days post-treatment [12].

The history of *H*. *longicornis* population movement shows that this tick has a propensity to increase its geographical range [3, 13]. Therefore, it is appropriate to recommend treatment of dogs that are traveling from known areas of presence into regions that are not yet known to have established tick populations. The high level of efficacy achieved following fluralaner administration indicates that this would be an appropriate treatment to administer to traveling dogs to reduce the potential that these animals could act as carriers for introduction of this tick into new areas.

## Conclusions

A single oral administration of 25–50 mg/kg fluralaner in a chewable tablet to dogs provides up to 114 days of post-treatment protection against challenge with *H*. *longicornis* ticks.

## Abbreviation

SFTS: Severe fever with thrombocytopenia syndrome
